# Percutaneous closure of simple congenital heart diseases under echocardiographic guidance

**DOI:** 10.1186/s40001-023-01398-8

**Published:** 2023-10-07

**Authors:** Ying Jiang, Fanyan Luo, Haisong Bu

**Affiliations:** The Department of Cardiovascular Surgery, Xiangya Hospital, Central South University, 87 Xiangya Road, Changsha, 410008 Hunan People’s Republic of China

**Keywords:** Congenital heart disease, Percutaneous closure, Echocardiographic, Transesophageal echocardiography

## Abstract

Congenital heart disease (CHD), birth defect with the highest incidence rates worldwide, and is mainly characterized by the abnormal internal structure of the heart or/and the anatomical structure of great vessels. In the past few decades, CHD repair surgery through standard median sternotomy incision combined with cardiopulmonary bypass (CPB) technology has been considered the gold standard for surgical correction of heart and great vessels. With the promotion and clinical application of interventional catheterization technology, transcatheter closure of CHD under radioactive radiation has gradually been recognized and applied. However, its radiation exposure and potential complications related to arteriovenous vessels still face challenges. In recent years, an increasing number of surgeons have explored new surgical procedures, for the safe and effective treatment of CHD, as far as possible to reduce surgical trauma, avoid radiation exposure, and improve the cosmetic effect. Therefore, on the premise of satisfactory exposure or guidance, how to integrate ultrasound and percutaneous interventional technology remained the focus of the exploration. This mini-review highlights and summarizes the signs of progress of ultrasound intervention in the last decade that have proven the effectiveness and operability of a well-established procedure for percutaneous closure of congenital heart diseases under echocardiographic guidance only. We discuss potential diseases that will benefit from this emerging procedure based on this progress. Owing to the crucial advantages played by this strategy in the treatment of CHD, better understanding and promotion of this less exploited field may contribute to the development of therapeutics targeting CHD, improve medical utilization rate, promote the optimization of medical resources, and ultimately achieve precise and efficient medical treatment.

## Introduction

Congenital heart disease (CHD), birth defect with the highest incidence rates worldwide, and is mainly characterized by the abnormal internal structure of the heart or/and anatomical structure of Great vessels [1, 2]. Based on clinical features, CHD can be broadly classified as simple CHD and complex CHD. Simple CHD is probably composed of four types of diseases, atrial septal defect (ASD), ventricular septal defect (VSD), patent ductus arteriosus (PDA), and pulmonary valve stenosis (PVS). At the same time, these four types of cardiovascular structural abnormalities are the most common forms of CHD and one of the major causes of disease morbidity or mortality in the world, causing a huge psychological burden to people and an increasing economic burden to society.

In the past few decades, CHD repair surgery through standard median sternotomy incision combined with cardiopulmonary bypass (CPB) technology has been considered the gold standard for surgical correction of heart and great vessels. However, this early golden standard surgical approach faces challenges such as postoperative discomfort and residual long scar incisions in the median incision [3, 4].

With the promotion and clinical application of interventional catheter technology, the transcatheter intervention program assisted by radiation is gradually applied to treat some simple CHDs and is gradually recognized by the Food and Drug Administration (FDA) under the long-term monitoring of effectiveness and safety. Meanwhile, this simple transcatheter CHD strategy is still a valuable alternative and is currently widely used in many developing countries (such as China and other countries) and developed countries, even becoming a mature standard treatment for simple CHD [5–9], but technology can be limited to low weight patients (with small blood vessel diameter) or poor vascular development, and there are problems such as repeated exposure to medical radiation that affect patient growth and development [10, 11]. In addition, due to the compression of the occluder on the surrounding cardiac tissue leading to atrioventricular block (AVBs), which may lead to serious complications such as patient death, there is still controversy and caution in promoting transcatheter closure devices for CHD in clinical practice [12, 13]. As patients’ expectations for treatment (effectiveness, safety, and non-radiation) become higher and higher, cardiac surgery, under the guidance of non-radiation transesophageal echocardiography (TEE) guided medical image technology, developed a minimally invasive pericardiac device occlusion technique through a small incision at the lower sternum. This technology still has shortcomings, including surgical trauma, sternal fracture, and incision scars [14–17].

**Fig. 1:**
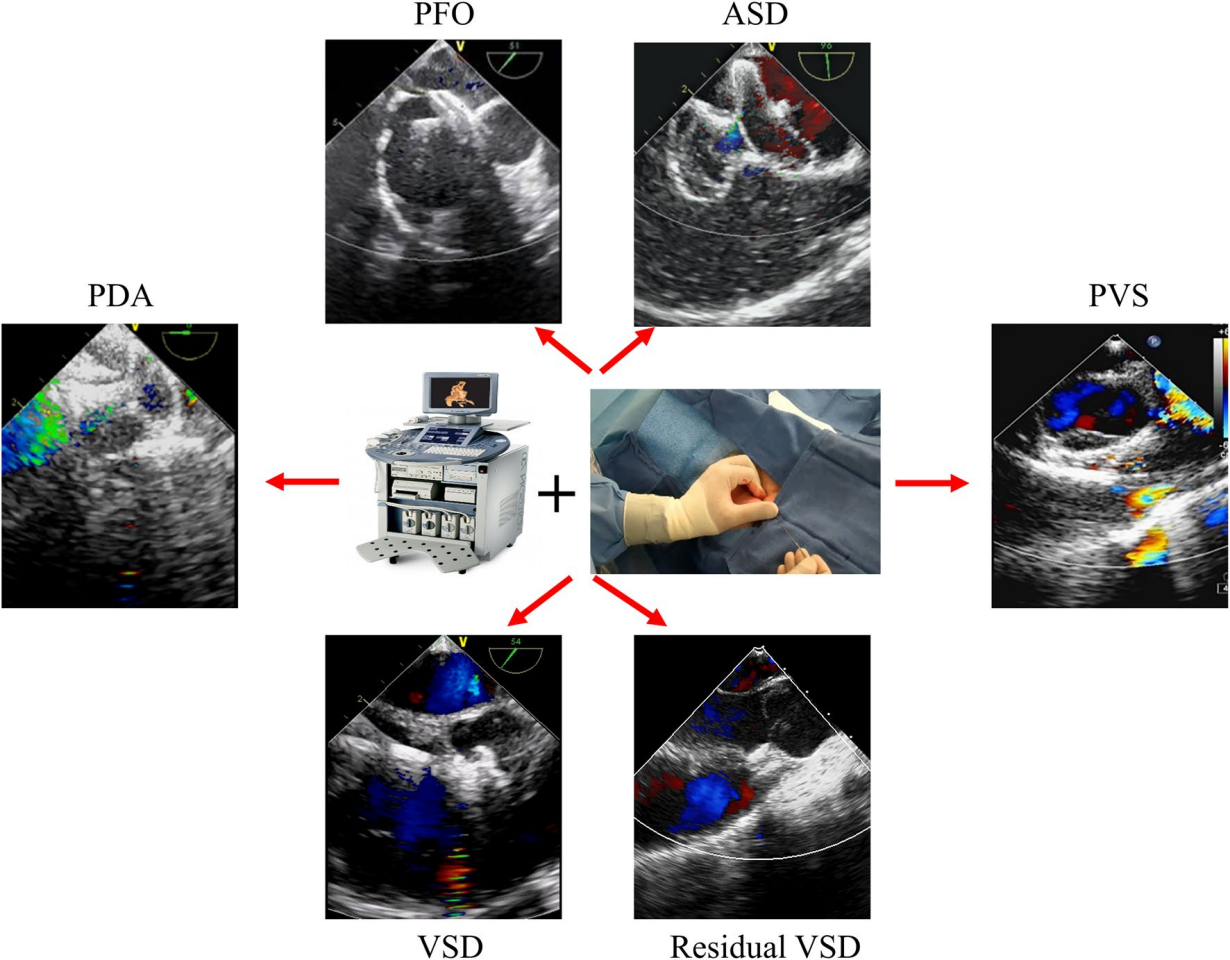
A well-established procedure for percutaneous closure of congenital heart diseases under echocardiographic guidance only. ASD: atrial septal defect, PFO: patent foramen ovale, VSD: ventricular septal defect, PDA: patent ductus arteriosus, PVS: pulmonary valve stenosis

With the popularization of echocardiography-guided technology, cardiac surgery combined with an interventional catheterization department has explored a new surgical method for the treatment of simple CHD, for the safe and effective treatment of CHD, as far as possible to reduce surgical trauma, avoid radiation exposure, and improve the cosmetic effect [18–20]. The research focus of many surgical operators is on exposure based on operability, clear command of color Doppler ultrasound, concealed areas or small puncture openings, and avoidance of operational and postoperative complications [21–23]. Therefore, the combination of ultrasound technology with fluoroscopy free and satisfactory guidance and percutaneous intervention technology without surgical trauma and incisions have been gradually promoted and applied. This mini-review highlights and summarizes the signs of progress of ultrasound intervention in the last decade that have proven the effectiveness and operability of a well-established procedure for percutaneous closure of congenital heart diseases under echocardiographic guidance only (Fig. 1). We discuss potential diseases that will benefit from this emerging procedure based on this progress. Owing to the crucial advantages played by this strategy in the treatment of CHD, better understanding and promotion of this less exploited field may contribute to the development of therapeutics targeting CHD, improve medical utilization rate, promote the optimization of medical resources, and ultimately achieve precise and efficient medical treatment.

## Echocardiography-guided percutaneous closure of simple CHDs

The development of cardiac intervention strategies is closely related to the advancement of computer imaging technology and clinical applications. Based on ultrasound technology, transcatheter intervention in simple CHD not only has less trauma, but also enables rapid recovery, saves medical costs, and shortens hospital stays. Many international ultrasound and heart disease guidelines [24, 25] point out that echocardiography plays a crucial role in the diagnosis of simple CHD diseases, evaluation of abnormal anatomical structures, selection of occlusive devices and delivery devices, and guidance on intraoperative and postoperative monitoring. Researchers have shown through research that a simple transcatheter therapy for CHDs based on TEE can reduce surgical procedure time, avoid radiation exposure risks, and improve mid to long-term postoperative prognosis, fully demonstrating the efficiency and safety of its approach [26].

## ASD and PFO

PFO is a common and persistent intracardiac anatomic abnormality, which has certain relevance to the occurrence of nervous system disease (such as cryptogenic stroke, transient ischemic attack, and migraine with aura among other clinical manifestations) [27, 28]. Epidemiological studies have shown that PFO diseases have a high incidence rate of approximately 15–20% [29, 30]. The causal relationship between the existence of PFO and the occurrence of neurological events has been explored by researchers, and clear treatment strategies have been developed [31, 32]. However, Carroll JD et al. [33] and Tobis JM et al. [34] reported that after PFO medical intervention for patients with migraine aura, their migraine symptoms were alleviated, which may be closely related to the risk of blocking the right to left micro embolism. What’s more, studies have confirmed a causal relationship between PFO and cryptogenic stroke, especially in elderly patients (> 55 years old) [35]. Interventional PFO closure used to be achieved through interventional therapy intervention under catheter fluoroscopy, and guidance and command under TEE [36]. With the rich clinical experience of interventional therapy, some experts in cardiac surgery or interventional cardiology can only complete PFO closure through fluoroscopy guidance, but the effectiveness and safety must be evaluated by TEE after surgery [33, 37, 38]. However, for some cases of PFO with complex anatomy, we believe that TEE support is needed for PFO closure. A comparative study by Paolo Scacciatella and colleagues [39] reported for the first time the use of esophageal echocardiography to guide PFO closure, and clarified that its approach can significantly reduce postoperative complications (residual shunt rate and valve regurgitation) and avoid adverse events such as radiation exposure. These are all based on clear image guidance and real-time dynamic command and adjustment of esophageal echocardiography.

Based on our heart center's mature and simple CHD occlusion technology and experience [22], we excluded neurogenic factors and only performed transcatheter PFO occlusion under the guidance of TEE, based on the evaluation of neurologists (Fig. 1). During the sealing process, we found that the difficulty lies in guiding the guide wire through the small PFO, and other operational processes are similar to ASD. When choosing a PFO occluder, attention should be paid to the size of the left atrium to avoid affecting the opening of the occluder or causing serious complications such as arrhythmia or left atrial rupture. Taken together, numerous researchers and our heart center experience (78 patients with a success rate of 100% and no postoperative adverse events) have shown that percutaneous TEE-guided PFO closure is a safe and reliable treatment method, and will be promoted and evaluated in global heart centers [40–43].

**Fig. 2:**
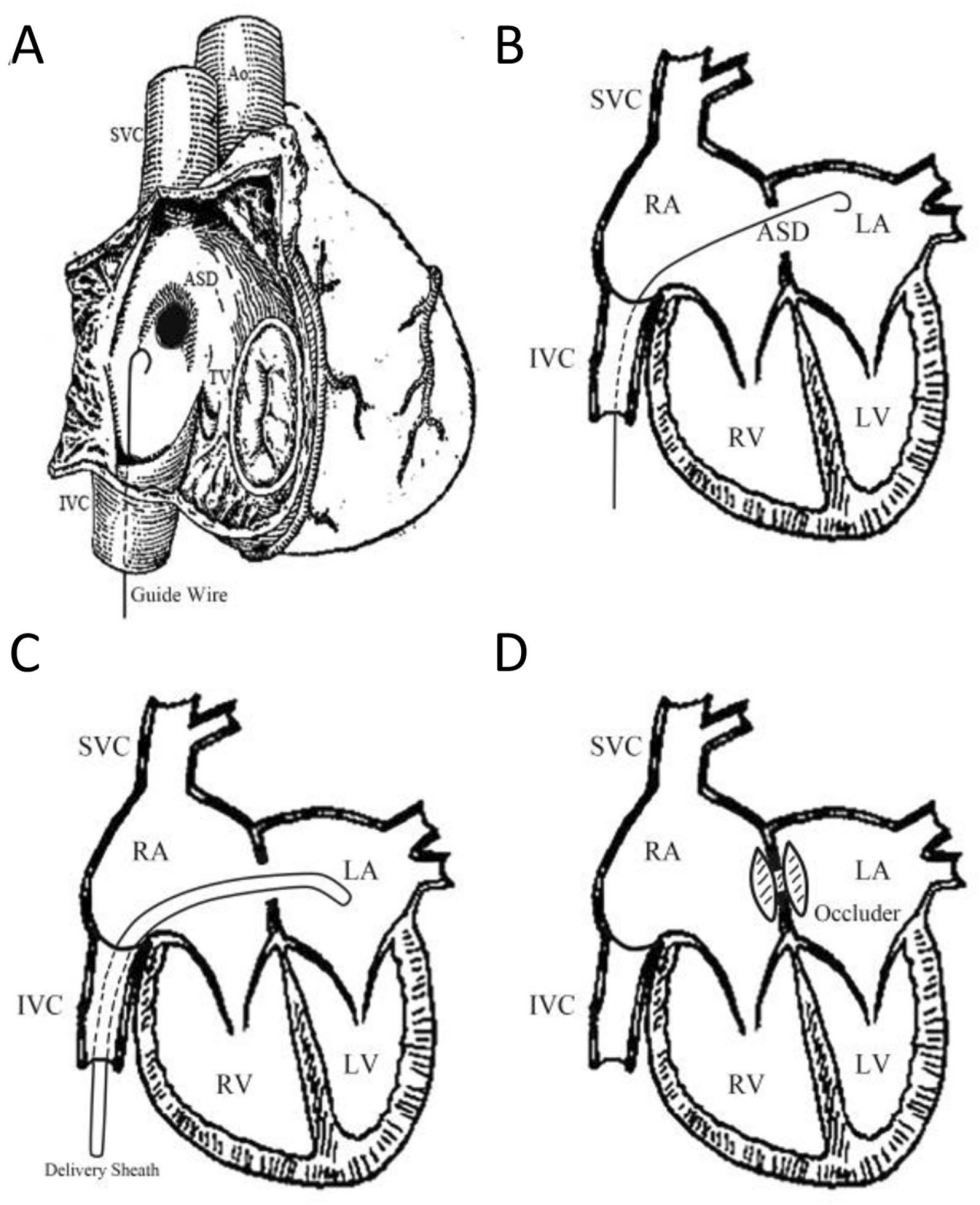
The steps of percutaneous device closure of ASD. **A** The guidewire was advanced into the RA. **B** The guidewire advanced through the ASD and into the LA. **C** The delivery sheath advanced into the LA guided by TEE. **D** The device was deployed. RA: right atrium; LA: left atrium; LV: left ventricle; RV: right ventricle; ASD: atrial septal defect; IVC: inferior vena cava; SVC: superior vena cava

ASD can be divided into four types: ostium primum, ostium secundum, sinus venosus, and unroofed coronary sinus and is the third common anatomical abnormality in CHD. Approximately 65% to 70% of patients with a secundum defect and about 50% of patients with primary ASD [7]. In 1976, King and his colleagues first reported the first case of successful closure of secundum ASD through a catheter under radiation fluoroscopy [44]. The revolutionary development and promotion of fluoroscopy and transcatheter treatment techniques led to fundamental changes in the treatment and management of ASD diseases [8]. With the change in the management mode of ASD, research has shown that fluoroscopy-guided transcatheter closure of secundum ASD has gradually replaced previous thoracotomy repair methods [7]. Based on this method, it not only has high efficiency and safety [45–47], but also has good visualization and tracking performance under fluoroscopy [22]. The only drawback is the presence of radiation exposure and vascular complications. However, radiation exposure in childhood is of more and more concern because children are more sensitive to radiation exposure than adults [48, 49], radiation covers most or even all parts of the body, and due to the unique characteristics of children, it may be necessary to repeatedly expose them to radiation [50, 51]. To avoid radiation exposure in transcatheter closure of ASD, a fluoroscopy­free technique under the guidance of echocardiography alone was first developed by Ewert and colleagues in 2000 [52, 53], though the study sample size was relatively small. Fortunately, in recent years, we have carefully studied the technology and improved the delivery system, and then established a standard procedure [22]. After that, the procedure time was shortened and no intervention­related complication occurred in subsequent patients. Thus, in the early stage of our heart center [22] demonstrated that using TEE technology for guiding transcatheter closure of secundum ASD is a safe and reliable strategy, and is routinely used in multiple cardiac centers such as ours, especially for children who cannot tolerate radiation or contrast agents (Fig. 2). Although it is a routine and feasible surgical plan, it is necessary to choose the appropriate occluder model based on the size of the left atrium during the surgery to avoid postoperative complications.

## VSD and residual VSD

Ventricular septal defect (VSD) refers to the direct abnormal defect of left and right ventricles, which accounts for the highest proportion of CHDs, of which 80% is classified as perimembranous VSD [54]. In the past decade, great progress has been made in the treatment and management of VSD.

**Fig. 3:**
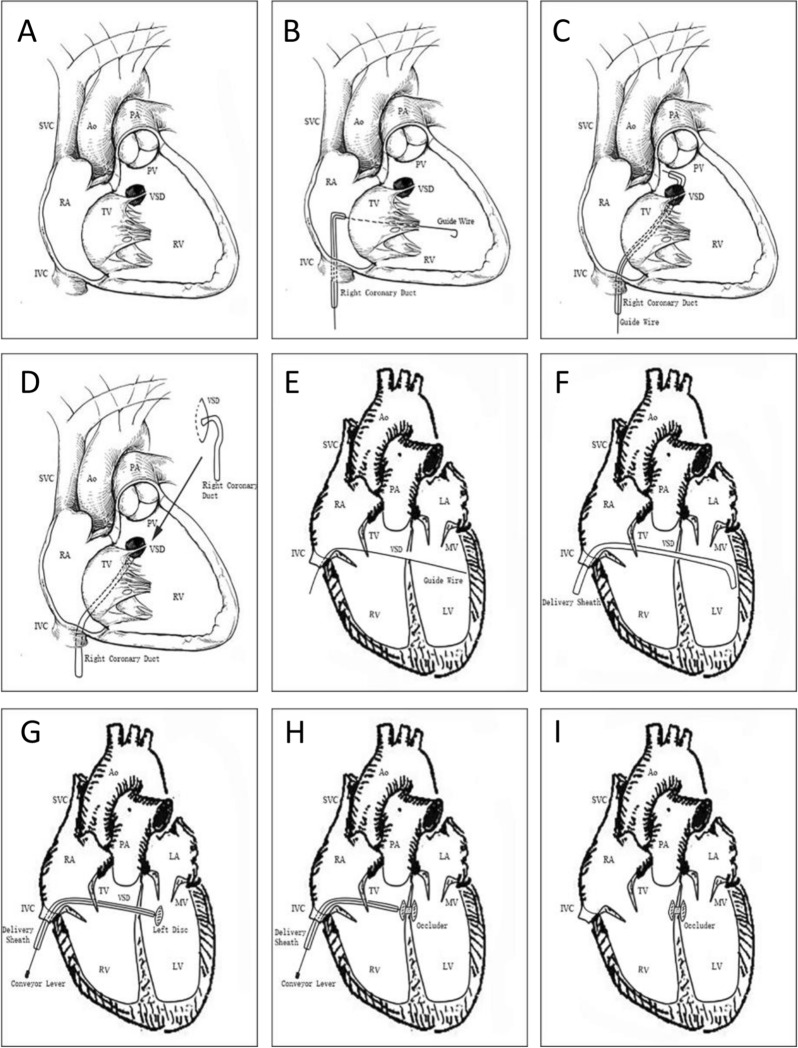
The steps of percutaneous device closure of perimembranous VSD. **A** The perimembranous VSD anatomical structure. **B** The guide-wire was introduced into the RV. **C** The tip of the sheath was advanced into the LV. **D** The sheath was advanced into the LV. **E** The guide-wire was introduced into the LV. **F** The delivery sheath advanced into the LV. **G** The left side of the disc was deployed. **H** The right side of the disc was deployed; **I** The device was deployed. RA: right atrium; LA: left atrium; LV: left ventricle; RV: right ventricle; VSD: ventricle septal defect; IVC: inferior vena cava; SVC: superior vena cava; PA: pulmonary artery

As a traditional gold standard treatment method, midline sternotomy and open heart surgery with cardiopulmonary bypass (CPB) have always been the only options for VSD repair; however, it is undeniable that there are multiple postoperative complications and residual long scar incisions during open chest surgery [4]. Under the guidance of fluoroscopy, the successful transcatheter closure intervention of VSD (muscle) promoted the promotion of its means, and the Food and Drug Administration approved the clinical application of this kind of surgery in 2007 [55]. Subsequently, based on experience accumulation, transcatheter closure of VSD became a research hotspot and gradually remained a good and mature alternative, especially widely promoted in developing countries such as China [56] and India [5, 6]; However, due to the unique location and high risk of postoperative arrhythmia of the occluder, approval has not yet been obtained in the United States [57]. Due to the limited development of peripheral blood vessels in low-weight children and the fixation of the delivery system, implementing transcatheter closure of VSD in such patients remains a current challenge [57]. To further meet the expectations of patients, fully reduce surgical trauma, and avoid radiation exposure, experts in Cardiac surgery and interventional cardiology, based on the advantages of esophageal echocardiography, popularized its combination for minimally invasive intervention in perimembranous VSD diseases, and gradually proved its safety and effectiveness [3, 14, 15, 58]. In addition, many previous researches [3, 12, 58] suggested among the selected VSD patients, there is no significant difference in effectiveness and safety among fluoroscopy-guided transcatheter closure, traditional direct vision repair, and mini-invasive periventricular device closure of VSDs, and they have a certain degree of substitutability [17]. To avoid surgical trauma, incision, and radiation exposure, Shouzheng Wang et al. [59] and Haisong Bu et al. [23] reported a novel strategy, which is guided by TEE to close the perimembranous VSD through the femoral vein, without the need for establishing arteriovenous loop, and radiation exposure, surgical trauma, and incision scars, which has excellent safety and feasibility (Fig. 3). Therefore, this novel procedure should be a promising alternative use in patients that has been widely used in China.

Residual shunting after VSD repair or occlusion is a common phenomenon and has been reported to account for 5–36% of all VSDs undergoing surgical repair [60]. Partial residual shunts have minimal impact on hemodynamics, and patients have no obvious symptoms. Based on the phenomenon of endothelialization after occluder placement, there may be a possibility of self-repair and closure [60, 61]. However, for VSD with obvious hemodynamic effects that need intervention, the main purpose of intervention is to prevent long-term related complications, such as pulmonary hypertension, Infectious endocarditis, and progressive aortic valve regurgitation caused by aortic valve prolapse [62]. At present, we still face some challenges, such as the high risk of reopening the chest to repair residual shunts, myocardial scar tissue, long-term extracorporeal circulation assistance, coagulation system sensitivity disorder, and causing physical and psychological trauma to patients once again [62]. Furthermore, if an arteriovenous ring is established through a percutaneous approach and residual VSD is occluded with a catheter occluder under fluoroscopy assistance, there are also some difficulties, such as vascular development issues, vascular complications, arrhythmias, and valve damage [63]. What's more, interventional catheterization methods for intervening in radiation exposure in CHD are particularly important for infants and children, who are in the growth and development stage and have not yet matured various organs, making them more sensitive to radiation and more harmful than adults [50, 51]. Considering these issues, Xuming Mo and colleagues [64] first proposed a new strategy for treating residual shunts after VSD repair in 2016, which involves transcatheter closure of residual shunts through chest wall intercostal puncture and successful clinical application. In addition, A long-term systematic study by Haisong Bu and colleagues [63] reported and applied this new treatment strategy in the heart center based on previous research scholars’ reports, which successfully occluded residual VSD through chest wall puncture under echocardiographic localization and guidance, with advantages such as no arteriovenous ring, no radiation risk, no need for CPB, and no incision scar. However, this new transthoracic closure strategy requires mature and accurate ultrasound localization and guidance to ensure the smooth implementation of intervention measures.

## PDA

**Fig. 4:**
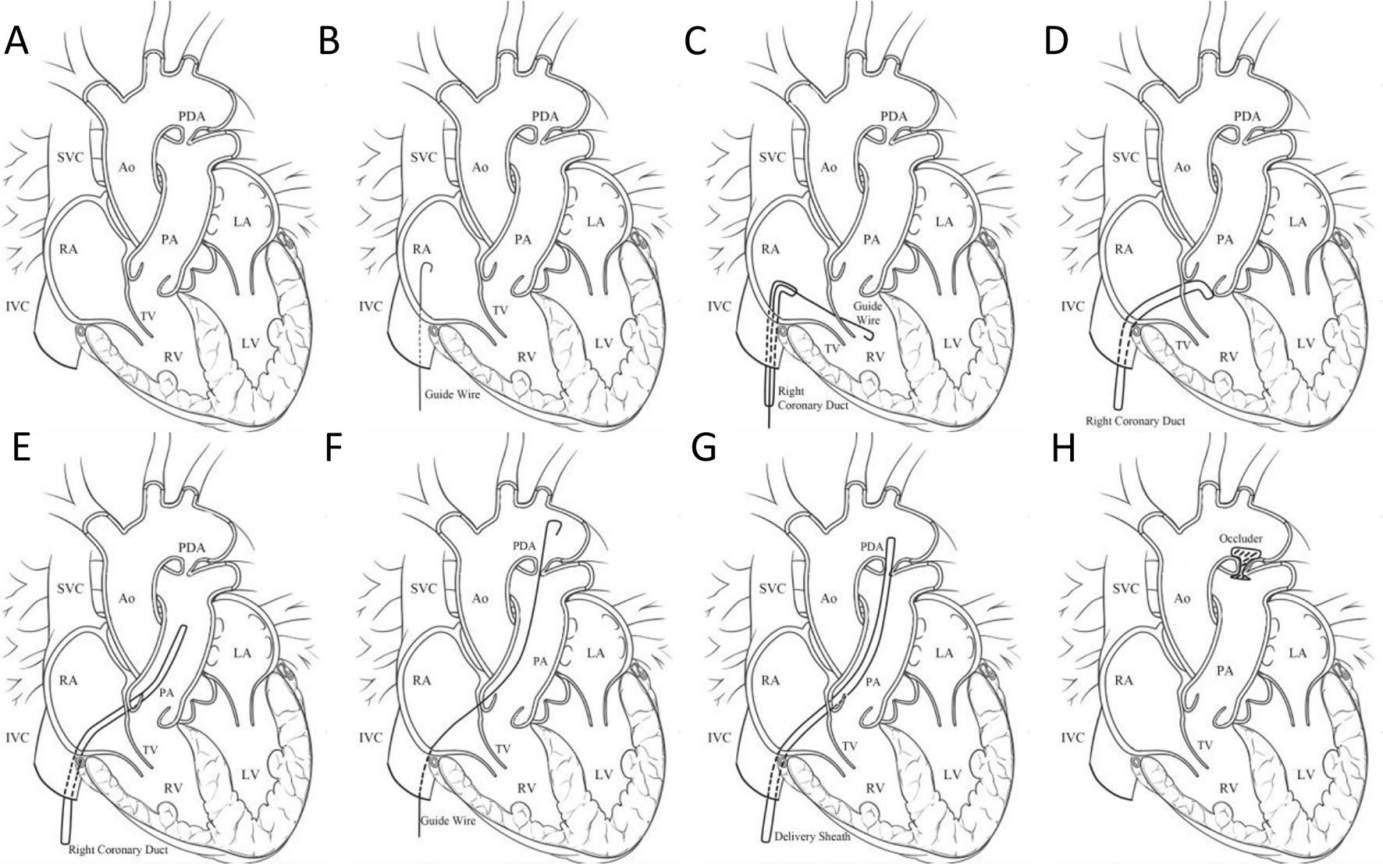
The steps of percutaneous device closure of PDA. **A** PDA anatomical structure. **B** The guidewire was advanced into the RA. **C** The guide-wire was introduced into the RV. **D** The sheath was advanced into the RV. **E** The sheath was advanced into the PA. **F** The guide-wire was introduced into the descending aorta. **G** The delivery sheath advanced into the descending aorta. **H** The device was deployed. RA: right atrium; LA: left atrium; LV: left ventricle; RV: right ventricle; PDA: patent ductus arteriosus; IVC: inferior vena cava; SVC: superior vena cava; PA: pulmonary artery

As an isolated lesion, PDA refers to a disease in which the blood vessels between the pulmonary artery and the descending aorta are not yet closed within a certain period after birth, resulting in left to right shunting and accounting for 8% to 10% of all CHDs [20]. In 1939, the PDA was successfully closed through surgical repair surgery. Subsequently, in 1967, fluoroscopy-guided transcatheter PDA closure was introduced and promoted, and significant changes and progress were made in the development of its delivery device and occlude [9]. In the following decades, fluoroscopy-guided transcatheter closure of PDA gradually replaced traditional open chest repair surgery, and its safety and operability were well confirmed [65, 66]. The standard method for transcatheter closure of PDA with Amplatzer Duct Occluder I (ADO-I) is to first establish a femoral arteriovenous loop, deliver the system into the descending aorta through the femoral vein under radiation guidance, and finally enter the pulmonary artery for occlusion intervention [67]. However, this complex operation will prolong the overall intervention time and pose an additional risk of peripheral vascular complications [68]. Recently, respective efforts have been made to either reduce radiation exposure or contrast agent usage [69, 70] or to avoid arterial access [71]. To develop a strategy to figure out all these issues simultaneously, based on long-term clinical experience, we have promoted a new intervention measure, which involves transcatheter PDA closure through the femoral vein pathway under TEE guidance. This new strategy has been promoted and proven to have good safety and operability in multiple heart centers (Fig. 4) [20, 72]. Taken together, this new strategy avoids the risk of radiation exposure, contrast agent allergies, and potential peripheral vascular complications, and can serve as an alternative intervention for PDAs of a certain size, especially for children in the growth and development stage. It is worth noting that when performing PDA occlusion, we should choose a reasonable occluder, and closely monitor the postoperative platelet condition, as well as the dosage and time of anticoagulant medication, to prevent the occurrence of related adverse events.

## PVS

**Fig. 5:**
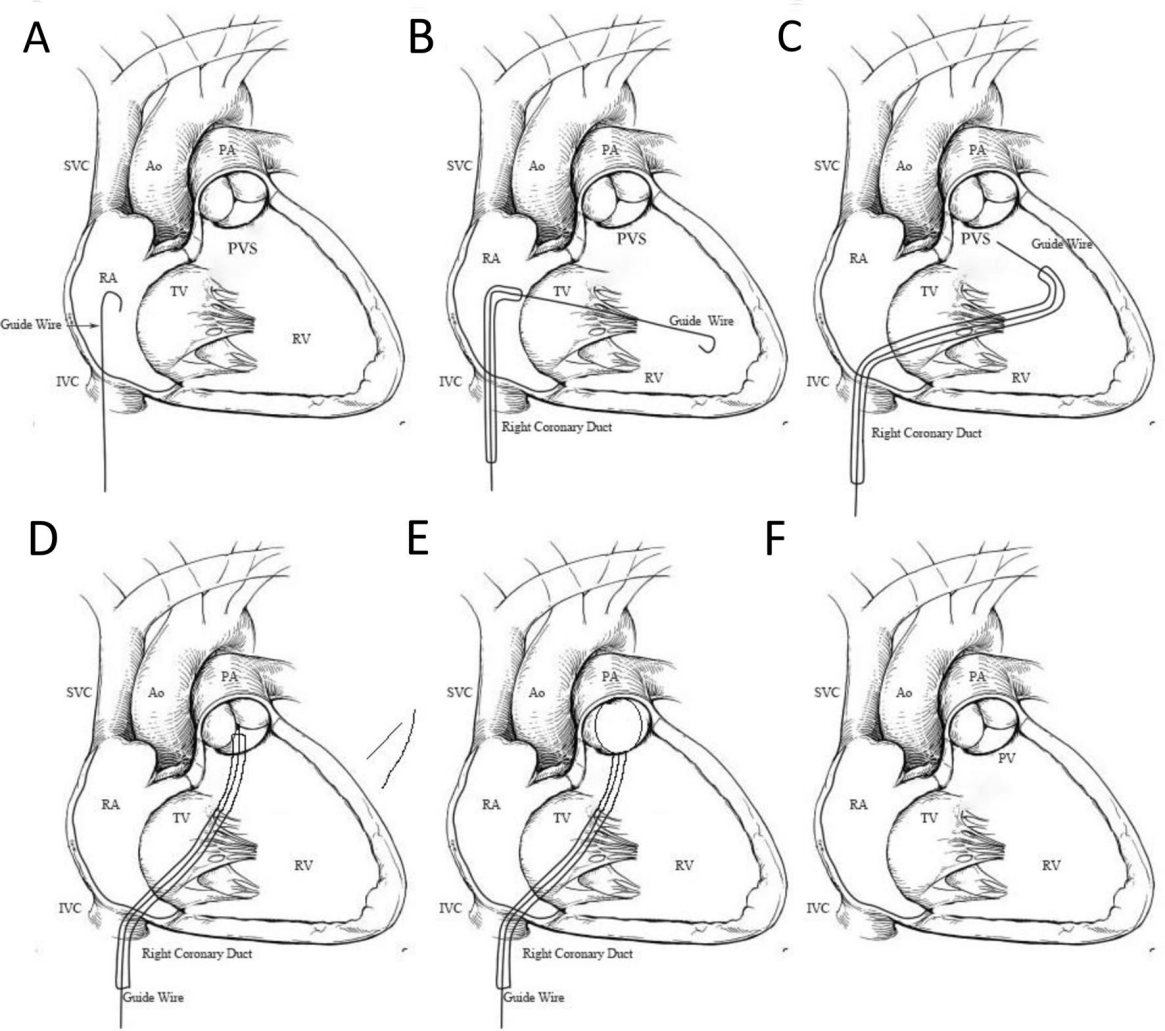
The steps of percutaneous expansion of PVS. **A** The guidewire was advanced into the RA. **B** The guide-wire was introduced into the RV. **C** The sheath was advanced into the RV. **D** The sheath was advanced into the PA. **E** Balloon dilation of narrowed PV. **F** Anatomical morphology after PVS balloon angioplasty. RA: right atrium; LA: left atrium; LV: left ventricle; RV: right ventricle; IVC: inferior vena cava; SVC: superior vena cava; PVS: pulmonary valve stenosis; PA: pulmonary artery

PVS refers to the narrowing of pulmonary artery valves caused by congenital developmental abnormalities, accounting for 8–10% of CHD. Its treatment methods mainly include thoracotomy under direct vision and balloon angioplasty. There is a risk of extracorporeal circulation and sternal sawing during pulmonary valvotomy, which can cause significant trauma to patients [73]. In 1982, Kan and other researchers successfully performed the first catheter-guided balloon pulmonary valvuloplasty under fluorescence radiation guidance. Compared with traditional open chest surgery, it has advantages such as less trauma, shorter hospitalization period, and less aesthetic and psychological burden, which has prompted this method to be quickly accepted by patients and promoted and applied in clinical practice [74, 75]. Conventionally, percutaneous balloon pulmonary valvuloplasty (BPV) is conducted under the guidance of fluoroscopy and the determination of arterial angiography. Although it has brought certain expectations and advantages to patients, the potential risks cannot be ignored, especially for children who are sensitive to radiation and contrast agents, Cardiac surgery who often perform surgical interventions, and patients with renal insufficiency or failure. Based on the existence of these potential risks, cardiac surgeons are encouraged to seek alternative imaging tools for guidance or develop a new intervention method to complete the shaping process. It is exciting to note that echocardiography has a high resolution for valves and good tracking performance. Fortunately, the use of echocardiography-guided interventional therapy for PVS has also become an important field. Research has shown that it cannot only avoid radiation from perspective and use contrast agents, but also more clearly display the anatomical structure of the heart, providing the best field of view for the surgeon [76–78]. A study by Shouzheng Wang and colleagues [79] conducted their first study in 2016, which showed that percutaneous BPV intervention was successfully performed only under the guidance of echocardiography, without the use of any other auxiliary imaging tools during the surgery, and echocardiography played a crucial role throughout the entire process. Follow-up showed that this new intervention strategy has good safety and effectiveness. Since then this well-established procedure has become popular in research and application [76], which has also been proven safe and effective through screening suitable patients and long-term follow-up in our center (Fig. 5). Thus, TEE-guided percutaneous BPV has been proven safe and effective and has gradually become an alternative treatment in patients. It is worth noting that this echocardiographic-guided percutaneous BPV requires strict selection of the size of the dilated balloon based on the patient’s vascular development. During surgery, it is necessary to prevent violent dilation of the pulmonary artery valve and strictly control the balloon formation time to avoid adverse events such as ischemia, hypoxia, and arrhythmia.

## Necessary of echocardiography-guided therapeutics for CHDs

The clear visualization of intervention operations is of great significance for ensuring the safety and operability of simple CHD treatment. Although the traditional method can provide some visual images and tracking guidance under the guidance of fluorescent radiation and the use of contrast agents, there is no doubt about the risks of this treatment strategy, especially for cardiac surgeons who often carry out surgical intervention, kidney dysfunction and children in the growth and development stage [80]. Research has shown that the use of contrast agents to induce kidney disease during medical testing or treatment is more common in clinical practice and is the main cause of 10% of patients with acute renal failure [81–83]. In addition, due to the unique characteristics of infants and children, high sensitivity to radiation, wide range of exposure areas, and potential for repetitive manipulation, as well as immature growth and development, the risk of long-term radiation exposure through catheter intervention is highly controversial and cannot be ignored [50, 51]. During infancy and childhood, prolonged exposure to low-dose radiation due to immature tissue structures or organs may lead to abnormal tissue and organ development, and even trigger cancer diseases, shortening the lifespan of the child [84]. Besides, it should be noted by medical workers that long-term low-dose exposure to radiation will be more likely to induce cancer, especially head and neck, and intracranial diseases, which will also damage the cardiovascular and cerebrovascular systems, especially accelerate vascular aging and increase the probability of arteriosclerosis [85, 86].

Based on the existence of these potential risks, cardiac surgeons are encouraged to seek alternative imaging tools for guidance or develop a new intervention method to complete the shaping process. In recent years, with the continuous improvement and promotion of echocardiography, it has been widely used as an imaging guide for percutaneous occlusion of simple CHD in various heart centers around the world, with real-time dynamics, and non-invasive and inexpensive advantages. Echocardiography plays an important role in the intervention and treatment of simple CHD, especially inpatient diagnosis, preoperative clarification of cardiac anatomy, surgical approach, selection of occluder model and delivery system, real-time intraoperative monitoring and guidance, and postoperative effectiveness evaluation [23, 87].

When guiding intervention with echocardiography, some materials will be routinely used to complete the surgery. Femoral vein access was achieved using a matched Fr sheath. A 0.038-inch bent stiff guidewire was advanced from the inferior vena cava via the right atrium into the superior vena cava. A 5Fr JR 4 diagnostic catheter was subsequently advanced into the right atrium over the guidewire which was then entered into the right ventricle via the catheter. Under the guidance of TEE, the operation can be completed by inserting the matching conveying system, occluder device, and balloon dilation system through the established trajectory.

**Fig. 6:**
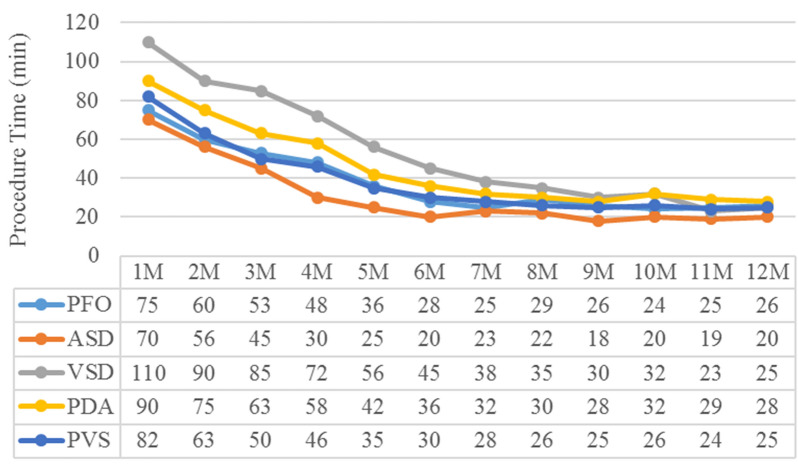
The learning curve of percutaneous treatment for different types of congenital heart disease. M: month, ASD: atrial septal defect, PFO: patent foramen ovale, VSD: ventricular septal defect, PDA: patent ductus arteriosus, PVS: pulmonary valve stenosis

Compared with traditional transthoracic echocardiography, TEE is located in the patient's esophagus, eliminating the influence of chest wall and lung tissue, enabling clearer images and clearer and more detailed cardiac anatomy. Real-time monitoring can achieve clarity and non-interference. In addition, TEE can display the entire pathway, including all blood vessels and tissue structures involved, to the operator without radiation throughout the entire process, and can provide three-dimensional images when appropriate, which can more vividly help the operator complete the occlusion operation smoothly. Echocardiogram guidance stenting ductus venous also plays an important role in intracardial anomalous pulmonary venous connection [88]. For cardiac surgeons in the early stage of TEE research, a certain amount of learning time and understanding of various sections and images are required. However, cardiac surgeons have the advantage of knowing the structure of the heart and blood vessels very well. Therefore, with the acquisition of operator experience, the operation time will be significantly shortened. As shown in our center (Fig. 6), the learning curve is very short for operators, especially for surgeons [20, 22, 23]. Taken together, based on the numerous advantages of TEE, simple CHD occlusion interventions under its guidance are gradually recognized by other heart centers or countries, and its indispensable advantages in the operation process are obvious.

## Limitations and future perspectives

However, there are certain shortcomings in the intervention of simple CHD guided by echocardiography. There are certain challenges for the surgeon, who not only have a thorough understanding of the anatomical structure of the heart and blood vessels, but also need to be very familiar with ultrasound guidance, ultrasound sections, and related parameters. Secondly, ultrasound-guided surgery is easily influenced by subjective factors, which further strengthens the test for surgical operators. The success of surgery is closely related to the experience of surgeons and ultrasound doctors. For TEE-guided intervention in simple CHDs surgery, echocardiogram doctors should have rich experience to prevent surgeons from having a blank in ultrasound professional knowledge, thus avoiding the inability to execute the program. In addition, ultrasound is prone to interference from tissues such as intrathoracic gas during guided intervention, which poses certain difficulties for surgery and may increase surgical time and anesthesia risks.

This mini-review highlights and summarizes the signs of progress of ultrasound intervention in the last decade that have proven the effectiveness and operability of a well-established procedure for percutaneous closure of congenital heart diseases under echocardiographic guidance only. We discuss potential diseases that will benefit from this emerging procedure based on these progresses. Owing to the crucial advantages played by this strategy in the treatment of CHD, better understanding and promotion of this less exploited field may contribute to the development of therapeutics targeting CHD, improve medical utilization rate, promote the optimization of medical resources, and ultimately achieve precise and efficient medical treatment. The TEE-guided percutaneous simple CHD treatment intervention method combines the advantages of Cardiac surgery, interventional radiology, and auxiliary medical departments, which has good safety and feasibility, and avoids the problems of low-dose radiation exposure, CPB, intraoperative and postoperative adverse events, and incision scars. One challenge faced by this new technology is how to apply it in low-weight or vascular dysplasia infants and young children. Notedly, Mini et al. [82, 83] reported echocardiogram guidance stenting of the aortic arch in very- low and extremely-low weight babies (600 g to 1300 g) with renal failure and showed that the interventions in such babies were feasible and can be done at the bedside to avoid transfer such babies to the cath lab. However, there is no experience in other CHDs. This is not only a challenge for the new technology, but also a challenge for traditional transcatheter closure interventions for simple CHDs.

Despite minor shortcomings, this well-established procedure has emerged as a powerful method for treating simple CHDs during the last decade. Its application is not limited to the establishment of known simple CHDs for therapeutic development, but should be extended to discovering new therapies and diseases. In the upcoming decade, this novel procedure promises to make a big splash in CHDs.

## Conclusions

Based on this brief review, we discussed the potential for simple CHDs to benefit from an emerging procedure. Owing to the crucial advantages played by this strategy in treating CHD, better understanding and promoting this less exploited field may contribute to the development of therapeutics targeting CHD. Furthermore, because this technology only depends on TEE and operator experience technology, it does not need to purchase expensive equipment, which is conducive to the promotion and application in grass-roots hospitals and Community hospitals, especially in remote and poor areas in China, ultimately improving medical utilization, promoting the optimization of medical resources, and ultimately achieving accurate and efficient medical services for patients.

## Data Availability

Not applicable.

## References

[1] Mustacchi P, Sherins RS, Miller MJ (1963). Congenital malformations of the heart and the great vessels. Prevalence, incidence, and life expectancy in San Francisco. JAMA..

[2] Bu H, Liu L, Hu S, Tan Z, Zhao T (2019). Targeted next-generation sequencing for research and diagnostics in congenital heart disease, and cleft lip and/or palate. Mol Med Rep.

[3] Hu S, Yang Y, Zhu Y, Wu Q, Muhoozi R, Wei S (2014). Experience with percardiac interventions for multiple congenital heart diseases in children. Interact Cardiovasc Thorac Surg.

[4] Rein JG, Freed MD, Norwood WI, Castaneda AR (1977). Early and late results of closure of ventricular septal defect in infancy. Ann Thorac Surg.

[5] Butera G, Carminati M, Chessa M, Piazza L, Micheletti A, Negura DG (2007). Transcatheter closure of perimembranous ventricular septal defects: early and long-term results. J Am Coll Cardiol.

[6] Carminati M, Butera G, Chessa M, De Giovanni J, Fisher G, Gewillig M (2007). Transcatheter closure of congenital ventricular septal defects: results of the European Registry. Eur Heart J.

[7] Geva T, Martins JD, Wald RM (2014). Atrial septal defects. Lancet.

[8] Opotowsky AR, Landzberg MJ, Kimmel SE, Webb GD (2008). Trends in the use of percutaneous closure of patent foramen ovale and atrial septal defect in adults, 1998–2004. JAMA.

[9] Baruteau AE, Hascoet S, Baruteau J, Boudjemline Y, Lambert V, Angel CY (2014). Transcatheter closure of patent ductus arteriosus: past, present and future. Arch Cardiovasc Dis.

[10] Omelchenko A, Gorbatykh Y, Voitov A, Zaitsev G, Bogachev-Prokophiev A, Karaskov A (2016). Perventricular device closure of ventricular septal defects: results in patients less than 1 year of age. Interact Cardiovasc Thorac Surg.

[11] Zeng XJ, Sun SQ, Chen XF, Ma XJ, Luo YH, Lim YP (2008). Device closure of perimembranous ventricular septal defects with a minimally invasive technique in 12 patients. Ann Thorac Surg.

[12] Bacha E (2015). New procedures, new complications, and new strategies to deal with them. J Thorac Cardiovasc Surg.

[13] Hu S, Yang Y, Wu Q, Zhao T (2015). Surgical treatment for patients with complete atrioventricular block after device closure of perimembranous ventricular septal defects. J Thorac Cardiovasc Surg.

[14] Quansheng X, Silin P, Zhongyun Z, Youbao R, Shengde L, Qian C (2009). Minimally invasive perventricular device closure of an isolated perimembranous ventricular septal defect with a newly designed delivery system: preliminary experience. J Thorac Cardiovasc Surg.

[15] Xing Q, Pan S, An Q, Zhang Z, Li J, Li F (2010). Minimally invasive perventricular device closure of perimembranous ventricular septal defect without cardiopulmonary bypass: multicenter experience and mid-term follow-up. J Thorac Cardiovasc Surg.

[16] Wu Q, Yang Y, Xu X, Gao L, Yang J, Wang X (2013). Echocardiography in mini-invasive surgical device closure of secundum atrial septal defects. Zhong Nan Da Xue Xue Bao Yi Xue Ban.

[17] You T, Yi K, Ding ZH, Hou XD, Liu XG, Wang XK (2017). Transcatheter closure, mini-invasive closure and open-heart surgical repair for treatment of perimembranous ventricular septal defects in children: a protocol for a network meta-analysis. BMJ Open.

[18] Vida VL, Padalino MA, Bhattarai A, Stellin G (2011). Right posterior-lateral minithoracotomy access for treating congenital heart disease. Ann Thorac Surg.

[19] Li G, Su J, Fan X, Li Z, Zhang J, Zhu Y (2015). Safety and efficacy of ventricular septal defect repair using a cosmetic shorter right lateral thoracotomy on infants weighing less than 5 kg. Heart Lung Circ.

[20] Zhang W, Gao L, Jin W, Wu Q, Hu S, Yang Y (2018). Echocardiography-guided percutaneous closure of patent ductus arteriosus without arterial access: feasibility and safety for a new strategy. Zhong Nan Da Xue Xue Bao Yi Xue Ban.

[21] An G, Zhang H, Zheng S, Wang W, Wu Q, Xing Q (2016). Minimally invasive surgical closure for doubly committed subarterial ventricular septal defects through a right subaxillary thoracotomy. Interact Cardiovasc Thorac Surg.

[22] Yang Y, Zhang W, Wu Q, Gao L, Jin W, Zhao T (2016). Transcatheter closure of atrial septal defects without fluoroscopy: a well-established procedure for alternative use in children. EuroIntervention.

[23] Bu H, Yang Y, Wu Q, Jin W, Zhao T (2019). Echocardiography-guided percutaneous closure of perimembranous ventricular septal defects without arterial access and fluoroscopy. BMC Pediatr.

[24] Zamorano JL, Badano LP, Bruce C, Chan KL, Goncalves A, Hahn RT (2011). EAE/ASE recommendations for the use of echocardiography in new transcatheter interventions for valvular heart disease. J Am Soc Echocardiogr.

[25] Silvestry FE, Cohen MS, Armsby LB, Burkule NJ, Fleishman CE, Hijazi ZM (2015). Guidelines for the echocardiographic assessment of atrial septal defect and patent foramen ovale: from the American Society of Echocardiography and Society for Cardiac Angiography and Interventions. J Am Soc Echocardiogr.

[26] Biner S, Perk G, Kar S, Rafique AM, Slater J, Shiota T (2011). Utility of combined two-dimensional and three-dimensional transesophageal imaging for catheter-based mitral valve clip repair of mitral regurgitation. J Am Soc Echocardiogr.

[27] Kent DM, Dahabreh IJ, Ruthazer R, Furlan AJ, Reisman M, Carroll JD (2016). Device closure of patent foramen ovale after stroke: pooled analysis of completed randomized trials. J Am Coll Cardiol.

[28] Rayhill M, Burch R (2017). PFO and migraine: is there a role for closure?. Curr Neurol Neurosci Rep.

[29] Meissner I, Khandheria BK, Heit JA, Petty GW, Sheps SG, Schwartz GL (2006). Patent foramen ovale: innocent or guilty? Evidence from a prospective population-based study. J Am Coll Cardiol.

[30] Hara H, Virmani R, Ladich E, Mackey-Bojack S, Titus J, Reisman M (2005). Patent foramen ovale: current pathology, pathophysiology, and clinical status. J Am Coll Cardiol.

[31] Di Tullio MR (2010). Patent foramen ovale: echocardiographic detection and clinical relevance in stroke. J Am Soc Echocardiogr..

[32] Rundek T (2008). PFO in stroke: a direct association or coincidence?. Eur J Neurol.

[33] Carroll JD, Saver JL, Thaler DE, Smalling RW, Berry S, MacDonald LA (2013). Closure of patent foramen ovale versus medical therapy after cryptogenic stroke. N Engl J Med.

[34] Tobis JM, Charles A, Silberstein SD, Sorensen S, Maini B, Horwitz PA (2017). Percutaneous closure of patent foramen ovale in patients with migraine: the PREMIUM trial. J Am Coll Cardiol.

[35] Homma S, Messe SR, Rundek T, Sun YP, Franke J, Davidson K (2016). Patent foramen ovale. Nat Rev Dis Primers.

[36] Fateh-Moghadam S, Steeg M, Dietz R, Bocksch W (2009). Is routine ultrasound guidance really necessary for closure of patent foramen ovale using the Amplatzer PFO occluder?. Catheter Cardiovasc Interv.

[37] Mangieri A, Godino C, Montorfano M, Arioli F, Rosa I, Ajello S (2015). PFO closure with only fluoroscopic guidance: 7 years real-world single centre experience. Catheter Cardiovasc Interv.

[38] Meier B, Kalesan B, Mattle HP, Khattab AA, Hildick-Smith D, Dudek D (2013). Percutaneous closure of patent foramen ovale in cryptogenic embolism. N Engl J Med.

[39] Scacciatella P, Meynet I, Giorgi M, Biava LM, Matranga I, Biasco L (2018). Angiography vs transesophageal echocardiography-guided patent foramen ovale closure: a propensity score matched analysis of a two-center registry. Echocardiography.

[40] Sperlongano S, Giordano M, Ciccarelli G, Bassi G, Malvezzi Caracciolo D'Aquino M, Del Giudice C (2022). Advances in percutaneous patent foramen ovale closure: from the procedure to the echocardiographic guidance. J Clin Med..

[41] Molnar AA, Abraham P, Merkely B, Nardai S (2022). Echocardiographic evaluation of atrial communications before transcatheter closure. J Vis Exp.

[42] Lan Q, Wu F, Ye X, Wang S, Zhong J (2023). Intracardiac vs. transesophageal echocardiography for guiding transcatheter closure of interatrial communications: a systematic review and meta-analysis. Front Cardiovasc Med..

[43] Achim A, Hochegger P, Kanoun Schnur SS, Moser L, Stark C, Pranevicius R (2023). Transesophageal echocardiography-guided versus fluoroscopy-guided patent foramen ovale closure: a single center registry. Echocardiography.

[44] King TD, Thompson SL, Steiner C, Mills NL (1976). Secundum atrial septal defect. Nonoperative closure during cardiac catheterization. JAMA.

[45] Everett AD, Jennings J, Sibinga E, Owada C, Lim DS, Cheatham J (2009). Community use of the amplatzer atrial septal defect occluder: results of the multicenter MAGIC atrial septal defect study. Pediatr Cardiol.

[46] Jones TK, Latson LA, Zahn E, Fleishman CE, Jacobson J, Vincent R (2007). Results of the U.S. multicenter pivotal study of the HELEX septal occluder for percutaneous closure of secundum atrial septal defects. J Am Coll Cardiol..

[47] Moore J, Hegde S, El-Said H, Beekman R, Benson L, Bergersen L (2013). Transcatheter device closure of atrial septal defects: a safety review. JACC Cardiovasc Interv.

[48] Johnson JN, Hornik CP, Li JS, Benjamin DK, Yoshizumi TT, Reiman RE (2014). Cumulative radiation exposure and cancer risk estimation in children with heart disease. Circulation.

[49] Andreassi MG, Ait-Ali L, Botto N, Manfredi S, Mottola G, Picano E (2006). Cardiac catheterization and long-term chromosomal damage in children with congenital heart disease. Eur Heart J.

[50] Allen HD, Driscoll DJ, Fricker FJ, Herndon P, Mullins CE, Snider AR (1991). Guidelines for pediatric therapeutic cardiac catheterization. A statement for health professionals from the Committee on Congenital Cardiac Defects of the Council on Cardiovascular Disease in the Young, the American Heart Association. Circulation.

[51] Bacher K, Bogaert E, Lapere R, De Wolf D, Thierens H (2005). Patient-specific dose and radiation risk estimation in pediatric cardiac catheterization. Circulation.

[52] Ewert P, Daehnert I, Berger F, Kaestner A, Krings G, Vogel M (1999). Transcatheter closure of atrial septal defects under echocardiographic guidance without X-ray: initial experiences. Cardiol Young.

[53] Ewert P, Berger F, Daehnert I, van Wees J, Gittermann M, Abdul-Khaliq H (2000). Transcatheter closure of atrial septal defects without fluoroscopy: feasibility of a new method. Circulation.

[54] Hoffman JI, Kaplan S (2002). The incidence of congenital heart disease. J Am Coll Cardiol.

[55] Holzer R, Balzer D, Cao QL, Lock K, Hijazi ZM (2004). Amplatzer muscular ventricular septal defect I: device closure of muscular ventricular septal defects using the amplatzer muscular ventricular septal defect occluder: immediate and mid-term results of a U.S. registry. J Am Coll Cardiol.

[56] Qin Y, Chen J, Zhao X, Liao D, Mu R, Wang S (2008). Transcatheter closure of perimembranous ventricular septal defect using a modified double-disk occluder. Am J Cardiol.

[57] Gu M, You X, Zhao X, Zheng X, Qin YW (2011). Transcatheter device closure of intracristal ventricular septal defects. Am J Cardiol.

[58] Hu S, Yang Y, Wu Q, Rwakaryebe M, Liu Z, Deng Y (2014). Results of two different approaches to closure of subaortic ventricular septal defects in children. Eur J Cardiothorac Surg.

[59] Wang S, Ouyang W, Liu Y, Zhang F, Guo G, Zhao G (2018). Transcatheter perimembranous ventricular septal defect closure under transthoracic echocardiographic guidance without fluoroscopy. J Thorac Dis.

[60] Dodge-Khatami A, Knirsch W, Tomaske M, Pretre R, Bettex D, Rousson V (2007). Spontaneous closure of small residual ventricular septal defects after surgical repair. Ann Thorac Surg.

[61] Gabriel HM, Heger M, Innerhofer P, Zehetgruber M, Mundigler G, Wimmer M (2002). Long-term outcome of patients with ventricular septal defect considered not to require surgical closure during childhood. J Am Coll Cardiol.

[62] Momma K, Toyama K, Takao A, Ando M, Nakazawa M, Hirosawa K (1984). Natural history of subarterial infundibular ventricular septal defect. Am Heart J.

[63] Bu H, Yang Y, Wu Q, Zhao T (2020). Percutaneous puncture closure of postoperative residual ventricular septal defects without radiation. Ann Thorac Surg.

[64] Mo X, Qi J, Zuo W (2016). Percutaneous punctured transcatheter device closure of residual shunt after ventricular septal defect repair. Case Rep Cardiol.

[65] Thanopoulos BD, Hakim FA, Hiari A, Goussous Y, Basta E, Zarayelyan AA (2000). Further experience with transcatheter closure of the patent ductus arteriosus using the Amplatzer duct occluder. J Am Coll Cardiol.

[66] Pass RH, Hijazi Z, Hsu DT, Lewis V, Hellenbrand WE (2004). Multicenter USA Amplatzer patent ductus arteriosus occlusion device trial: initial and one-year results. J Am Coll Cardiol.

[67] Faella HJ, Hijazi ZM (2000). Closure of the patent ductus arteriosus with the amplatzer PDA device: immediate results of the international clinical trial. Catheter Cardiovasc Interv.

[68] Vijayalakshmi IB, Chitra N, Praveen J, Prasanna SR (2013). Challenges in device closure of a large patent ductus arteriosus in infants weighing less than 6 kg. J Interv Cardiol.

[69] Tanidir IC, Guzeltas A, Ergul Y, Ozturk E, Ozyilmaz I, Odemis E (2014). Transcatheter patent ductus arteriosus closure with echocardiographic guidance: can radiation exposure be reduced?. Turk Kardiyol Dern Ars.

[70] Sivakumar K, Bhagyavathy A, Gnanapragasam F (2009). Closure of large patent ductus arteriosus in renal failure under echocardiographic guidance without use of radiographic contrast media. Congenit Heart Dis.

[71] Garg G, Srivastava A, Tyagi H, Reddy SP, Radha AS (2013). Transcatheter device closure of patent ductus arteriosus without arterial access–single institution experience. Indian Heart J.

[72] Pan X, Ouyang W, Li S, Guo G, Liu Y, Zhang D (2015). Safety and efficacy of percutaneous patent ductus arteriosus closure solely under thoracic echocardiography guidance. Zhonghua Xin Xue Guan Bing Za Zhi.

[73] Rao PS (2007). Percutaneous balloon pulmonary valvuloplasty: state of the art. Catheter Cardiovasc Interv.

[74] Kan JS, White RI, Mitchell SE, Gardner TJ (1982). Percutaneous balloon valvuloplasty: a new method for treating congenital pulmonary-valve stenosis. N Engl J Med.

[75] Maostafa BA, Seyed-Hossien M, Shahrokh R (2013). Long-term results of balloon pulmonary valvuloplasty in children with congenital pulmonary valve stenosis. Iran J Pediatr.

[76] Deng RD, Zhang FW, Zhao GZ, Wen B, Wang SZ, Ou-Yang WB (2020). A novel double-balloon catheter for percutaneous balloon pulmonary valvuloplasty under echocardiographic guidance only. J Cardiol.

[77] Patel H, Raisinghani A, DeMaria A (2018). Echocardiography in transcatheter structural heart disease interventions. Prog Cardiovasc Dis.

[78] Bu H, Gao L, Zhang W, Wu Q, Jin W, Tang M (2017). Application of perimembranous ventricular septal defects closure solely by femoral vein approach under transesophageal echocardiography guidance. Zhong Nan Da Xue Xue Bao Yi Xue Ban.

[79] Wang SZ, Ou-Yang WB, Hu SS, Pang KJ, Liu Y, Zhang FW (2016). First-in-human percutaneous balloon pulmonary valvuloplasty under echocardiographic guidance only. Congenit Heart Dis.

[80] Holzer RJ, Gauvreau K, Kreutzer J, Trucco SM, Torres A, Shahanavaz S (2012). Safety and efficacy of balloon pulmonary valvuloplasty: a multicenter experience. Catheter Cardiovasc Interv.

[81] Potekhin NP, Nikitin AV, Evsiukov KB, Klopotskii SA, Zaitseva EA (2009). Progression prevention, diagnosis, and treatment of contrast agent-induced nephropathy. Voen Med Zh.

[82] Mini N, Zartner PA, Schneider MBE (2022). Stenting of critical aortic coarctation in neonates between 600 and 1350 g. Using a transfemoral artery approach. A single center experience. Front Cardiovasc Med.

[83] Mini N, Zartner PA, Sabir H, Suchowerskyj P, Schneider MBE (2023). Echocardiogram-guided stenting of a critical aortic coarctation in an extremely low weight preterm infant. JACC Case Rep.

[84] Dorfman AL, Fazel R, Einstein AJ, Applegate KE, Krumholz HM, Wang Y (2011). Use of medical imaging procedures with ionizing radiation in children: a population-based study. Arch Pediatr Adolesc Med.

[85] Roguin A, Goldstein J, Bar O, Goldstein JA (2013). Brain and neck tumors among physicians performing interventional procedures. Am J Cardiol.

[86] Andreassi MG, Piccaluga E, Gargani L, Sabatino L, Borghini A, Faita F (2015). Subclinical carotid atherosclerosis and early vascular aging from long-term low-dose ionizing radiation exposure: a genetic, telomere, and vascular ultrasound study in cardiac catheterization laboratory staff. JACC Cardiovasc Interv.

[87] Patrianakos AP, Zacharaki AA, Skalidis EI, Hamilos MI, Parthenakis FI, Vardas PE (2017). The growing role of echocardiography in interventional cardiology: the present and the future. Hellenic J Cardiol.

[88] George RS, Lozier JS, Bocks ML (2023). Palliative stenting of the venous duct in a premature neonate with obstructed infradiaphragmatic total anomalous pulmonary venous connection. Cardiol Young.

